# Do dental students need sonography training? A prospective observational study

**DOI:** 10.1186/s12909-025-07186-8

**Published:** 2025-04-23

**Authors:** Johannes Matthias Weimer, Maximilian Rink, Alexa Lippe, Lisa Zöll, Julian Künzel, Liv Lorenz, Christoph Sproll, Holger Buggenhagen, Lukas Müller, Lukas Pillong, Julia Weinmann-Menke, Anke Hollinderbäumer, Bilal Al-Nawas

**Affiliations:** 1 Rudolf Frey Learning Clinic, University Medical Centre of the Johannes Gutenberg University Mainz, Langenbeckstraße 1, 55131 Mainz, Germany; 2 Department of Medicine I and Center for Immunotherapy, University Medical Centre of the Johannes Gutenberg University Mainz, Mainz, Germany; 3 Department of Otorhinolaryngology, Head and Neck Surgery, University Hospital Regensburg, Regensburg, Germany; 4 Department of Oral and Maxillofacial Surgery - Facial Plastic Surgery, University Medical Center of the Johannes Gutenberg-University of Mainz, Mainz, Germany; 5 Polyclinic for Periodontology and Tooth Preservation, University Medical Center of the Johannes Gutenberg University Mainz, Mainz, Germany; 6 Department of Radiation Oncology and Radiotherapy, University Medical Center of the Johannes Gutenberg University Mainz, Mainz, Germany; 7 Department of Oral and Maxillofacial Surgery, University Hospital Düsseldorf, Heinrich-Heine- University Düsseldorf, Düsseldorf, Germany; 8 Department of Diagnostic and Interventional Radiology, Mainz University Hospital, Mainz, Germany; 9 Department for Otorhinolaryngology and Head- and Neck-Surgery, Saarland University Medical Center, Kirrbergerstraße 100, 66421 Homburg, Germany

**Keywords:** Dental education, Ultrasonography, Curriculum, Needs assessment, Diagnostic imaging

## Abstract

**Introduction:**

Sonography is a key diagnostic tool in oral and maxillofacial surgery and complements other imaging methods such as computer tomography or X-rays. While X-ray courses are integral to dentistry students’ training, ultrasound diagnostics have not been integrated into undergraduate and postgraduate training. This study investigates whether there is a demand for undergraduate sonography training among dental students.

**Methods:**

An online questionnaire was developed by a team of experts (dentistry, maxillofacial surgery, otorhinolaryngology, radiology, and didactics) based on the “Checklist for Reporting Results of Internet E-Surveys (Cherries)”. Multiple items addressed several topics using dichotomous (“yes”/ “no”), free text, and 7-level Likert answering formats (1 = not at all; 7 = fully and entirely). These included “personal data”, “previous experience”, “demand for ultrasound diagnostics in an undergraduate degree program”, “desired topics of ultrasound training”, “teaching methods”, and “study materials”. Dentistry students completed the questionnaire in the winter semester of 2023/24. The questionnaire’s validity was assessed using factor analysis, reliability testing (Cronbach’s Alpha, KR-20), and item discrimination.

**Results:**

129 dental students participated (74% female). Many respondents (approx. 90%) were in higher semesters (6–10 semesters) and stated that they had not yet had any contact with ultrasound diagnostics (75%) and had not performed an ultrasound examination as yet (97%). The overall demand for ultrasound training was high (5.8 ± 1.3), particularly for the mandibular joint (6.3 ± 1.1), parotid gland (6.2 ± 1.0), submandibular gland, sublingual gland (6.1 ± 1.1), and floor of the mouth and tongue (5.9 ± 1.4) topics. Concerning the teaching methods and teaching materials, “practical training on a proband” (82%), and the use of “teaching scripts” (85%), “video instructions” (74%), “digital pathological experts” (66%), “e-learning” (62%), and “blended learning” (52%) were most desired. The questionnaire demonstrated high reliability (Cronbach’s Alpha 0.93–0.95), strong factor structure (84% and 64% variance explained), and effective item discrimination.

**Conclusion:**

The data suggest a demand for undergraduate ultrasound training in dentistry. A practice-oriented, digitally supported training should be developed and implemented.

**Clinical trial number:**

Not applicable.

**Supplementary Information:**

The online version contains supplementary material available at 10.1186/s12909-025-07186-8.

## Introduction

Imaging procedures play a central role in clinical practice, especially in oral and maxillofacial surgery and dentistry [1]. Diagnosis, treatment, and follow-up of many diseases in these specialties can be aided by computer tomography (CT), cone beam CT (DVT), magnetic resonance imaging (MRI), conventional X-rays, and sonography [1, 2]. For example, X-rays (e.g., dental films or panoramic radiographs) are used for the imaging assessment of endodontics, periodontium, and jaw conditions. At the same time, a DVT also offers a three-dimensional representation of the tooth, jaw, and bone structures and is particularly helpful in implantology and the assessment of fractures [1–3]. MRI, conversely, offers excellent contrast for assessing soft tissue and the mandibular joint and detecting tumors and inflammatory processes in the head and neck area [1, 4].

In addition to these imaging procedures, sonography is a valuable imaging procedure [5–16]. Sonography allows for non-invasive, radiation-free real-time imaging and, thus, an assessment of the mandibular joints, salivary glands, the floor of the mouth, and the tongue. It may be used to diagnose soft tissue changes in the head and neck area, on the lips, and the oral mucosa, as well as for aftercare of dental implants and sometimes for detecting fractures. Sonography can be effectively used to detect cysts, tumors, and abscesses in the head and neck area and, for example, aiding in the differentiation between abscesses, cystic lesions, and inflammatory swellings. It allows real-time assessment of fluid collections, vascular structures, and echotexture, supporting early diagnosis and guiding targeted treatment [5–14, 17, 18].

Sonography has other exciting potential applications in dentistry, not least due to the technical development of ultra-high-frequency ultrasound probes that make it possible to visualize intraoral structures at high resolutions, such as the odontogenic cutaneous sinus tracts [14, 17, 18]. However, despite the importance of sonography in maxillofacial surgery and these potential advantages in dental practice, undergraduate training in both sonography in general and head and neck sonography, in particular, is often limited to medical students and rarely part of dentistry degree programs [19, 20]. Head and neck sonography training could offer numerous advantages for dental students, as has already been shown for medical students in several aspects:

- A better understanding of anatomy and a general understanding of cross-sectional images [21–23].
- Expansion of diagnostic capabilities and improvement of quality of care for patients [24, 25].
- Strengthening of self-confidence and competence in dealing with imaging procedures [21, 25, 26].
- More understanding of the relationships between imaging procedures and their advantages and disadvantages, including limitations [17, 21, 27].
- Improvement of interdisciplinary collaboration with colleagues from radiology, oral and maxillofacial surgery, and otolaryngology [24].

### Research problem and aim of the study

Ultrasound training is increasingly integrated into undergraduate medical training and postgraduate education and is highly desired by medical students and teachers [28]. Training concepts, including innovative teaching methods and examination formats for postgraduates [5, 29–35] and undergraduates in medicine [36–41], have already been published specifically for head and neck sonography. Despite the growing literature on the clinical application of sonography in dentistry [6–13, 24, 42, 43] and maxillofacial surgery [16], ultrasound training remains rudimentary or absent from most dentistry degree programs [20, 44]. Previous studies have shown that integrating ultrasound techniques into the clinical training of dentists could have numerous benefits, including improved diagnostic accuracy and increased understanding of soft tissue anatomy [20]. In-depth training could reduce the hesitation to use ultrasound in daily practice and expand diagnostic possibilities for dentists. However, there is currently no specific head and neck sonography training for dentistry students at German universities. This gap in training and lack of specifications of national competency-based learning objectives should be addressed to improve diagnostic possibilities and the training thereof [43–45]. Guided by the core principles of Kern´s Six-Step Approach, this study conducts a needs assessment among dentistry students at a German university as a preliminary step toward curricular development [46]. The collected data is intended to provide information about the demand among prospective dentists for undergraduate head and neck sonography training and which specific topics and teaching methods/study materials are preferred. Recommendations and approaches for implementing head and neck sonography training for dentistry students are subsequently discussed [46]. These recommendations should aid in developing practice-oriented and effective training curricula meeting students’ needs and modern dentistry’s requirements.

## Methods

### Study design, development, and implementation of the demand analysis

This prospective observational study was conducted at a German university clinic [47]. Based on the Kern-cycle, the problem identification and general needs assessment, including the attitude regarding future head and neck sonography curricula for dentists, was investigated with a targeted needs assessment [46]. The digital evaluation was designed according to the “checklist for reporting on internet e-surveys” (Cherries), which resulted in cooperation between dentists, otolaryngologists, radiologists, and didactic experts and psychologists [48]. Supplement 1 + 2 provides a detailed overview of the development of the survey, including the original survey questions. A pilot test (*n* = 10) was conducted to optimize structure and clarity. The questionnaire was performed in the winter semester of 2023/2024 (December 2023 to February 2024) and communicated to the students in lectures, on social media, and via e-mail by the dean’s office. Participation was possible using all standard devices (computer, tablet, and mobile phone). The evaluation was accessed via a link or by scanning a QR code. Dentistry students of all semesters who completed the assessment were included. To ensure data integrity, measures were implemented to prevent multiple submissions, and only fully completed surveys were included in the final analysis. Expert opinions were considered to assess content validity. Further instruments for determining reliability and validation can be found in the section below.

The primary endpoints are determining the demand for head and neck sonography training and the preferences for specific subjects. Secondary endpoints include the preferred teaching methods and study materials, the students’ pre-experiences and previous knowledge of ultrasound diagnostics, and a demographic analysis of the participants to identify relevant confounding factors. Strategies and recommendations for implementing training regarding “Goals and Objectives”, “Educational Strategies”, “Implementation”, and “Evaluation and Feedback” were derived [46].

In addition to the collection of “personal data” and “previous experiences in imaging procedures”, the demand for “integration of ultrasound diagnostics in the degree program” and preferences regarding “topics of an ultrasound training”, “teaching methods”, and “study materials” were recorded. Multiple items were assessed using a 7-point Likert answering format (1 = not up to 7 = fully and entirely), dichotomous questions (yes/no), and free text questions. Certain items, such as questions on possible ultrasound teaching relevant to “salivary glands” and “temporomandibular joint”, were included in the survey after observing their prominence in national dental education competency frameworks [44, 45]. Ethical approval was granted by the Ethics Commission of the Saarland Medical Association (ID: 228/23), and informed consent was obtained at the beginning of the survey.

### Data collection and statistics of the survey analysis, reliability and validity

Data was collected using the survey and test tool LimeSurvey (LimeSurvey GmbH, Germany). All data were saved with Microsoft Excel (Version 16.0). Statistical analyses were performed in Rstudio (Rstudio Team [2020]. Rstudio: Integrated Development for R. Rstudio, PBC, http://www.rstudio.com, last accessed on 20 04 2024) with R 4.0.3 (A Language and Environment for Statistical Computing, R Foundation for Statistical Computing, http://www.R-project.org; last accessed on 20 04 2024). Where possible, a main scale score was made from the average of the subscale scores. Binary and categorical baseline variables are given as absolute numbers and percentages. Continuous data are presented as median and interquartile range (IQR) or mean and standard deviation (SD). Categorical variables were compared using the chi-squared test, and continuous variables were compared using the t-test or the Mann-Whitney-U-test. These tests were also used to calculate the influence of the factors on the interest in sonography education. In addition, parametric (ANOVA) and non-parametric (Kruskal–Wallis) analyses of variance were performed, followed by pairwise post hoc tests (t-tests or Mann–Whitney U tests), as appropriate. Finally, a multivariate linear regression model was produced to compare the influence of individual factors. P-values < 0.05 were considered statistically significant.

A series of more statistical analyses were conducted to assess the reliability and validity of the questionnaire. The internal consistency of the questionnaire was measured using Cronbach’s Alpha for Likert-scale items and Kuder-Richardson-20 (KR-20) for dichotomous (yes/no) items. Cronbach’s Alpha was calculated separately for different subscales to determine the internal consistency of related items, while KR-20 was used to evaluate the reliability of dichotomous questions. An exploratory factor analysis (EFA) was conducted using the principal axis factoring (minimum residuals) method with Varimax rotation to examine the underlying structure of the questionnaire. The suitability of the data for factor analysis was assessed using the Kaiser–Meyer–Olkin (KMO) measure of sampling adequacy, where values above 0.80 indicate good suitability.

Additionally, Bartlett’s test of sphericity was applied to verify whether the correlation matrix was appropriate for factor extraction. Factors were retained based on eigenvalues greater than 1 and interpretability criteria, with factor loadings above 0.4 considered meaningful. Model fit was assessed using the Tucker-Lewis Index (TLI) and the Root Mean Square Error of Approximation (RMSEA). A TLI value above 0.90 indicated a good model fit, while RMSEA values below 0.08 were interpreted as acceptable. Lastly, an item analysis was conducted to assess the discrimination power of individual items using point-biserial correlations, with values exceeding 0.4 indicating good item discrimination. Items were reviewed for redundancy and theoretical relevance, ensuring that only the most informative items were retained.

## Results

### Sample and baseline characteristics

A total of 129 questionnaires were analyzed (Table 1). The average processing time of the questionnaire was 219 ± 91 s. Many participants were female (74%) and in the sixth semester or higher (92%). Most respondents already had experience with X-ray diagnostics (98%) as part of their university training, but only a few with sonography (25%). In addition, 97% stated that they had not previously performed an independent head and neck sonography. Overall, the respondents had encountered and used sonography significantly less often than other imaging procedures (*p* < 0.001).

**Table 1:** Sample baseline parameters

Item	
Sample size (n)	129
Age in years (mean ± SD)	25 ± 4.1
Sex	n [%]
female;	95 [74]
male	34 [26]
Semester	
1	4[3]
2	1 [1]
3	0 [0]
4	1 [1]
5	4 [3]
6	29 [23]
7	33 [26]
8	24 [19]
9	5 [4]
10	28 [22]
X-Ray in undergraduate programme	
Yes	126 [98]
No	3 [2]
CT in undergraduate programme	
Yes	77 [60]
No	52 [40]
MRI in undergraduate programme	
Yes	65 [50]
No	64 [50]
Sonography in undergraduate programme	
Yes	33 [26]
No	96 [74]
DVT in undergraduate programme	
Yes	99 [77]
No	30 [23]
Head and neck ultrasounds observed	
Yes	46 [36]
No	83 [64]
Head and neck ultrasound performed	
Yes	4 [3]
No	125 [97]

sd = standard deviation, Ct = computed tomography, mri = magnetic resonance imaging, dvt = cone beam Ct

### Results of the needs assessment

**Fig. 1:**
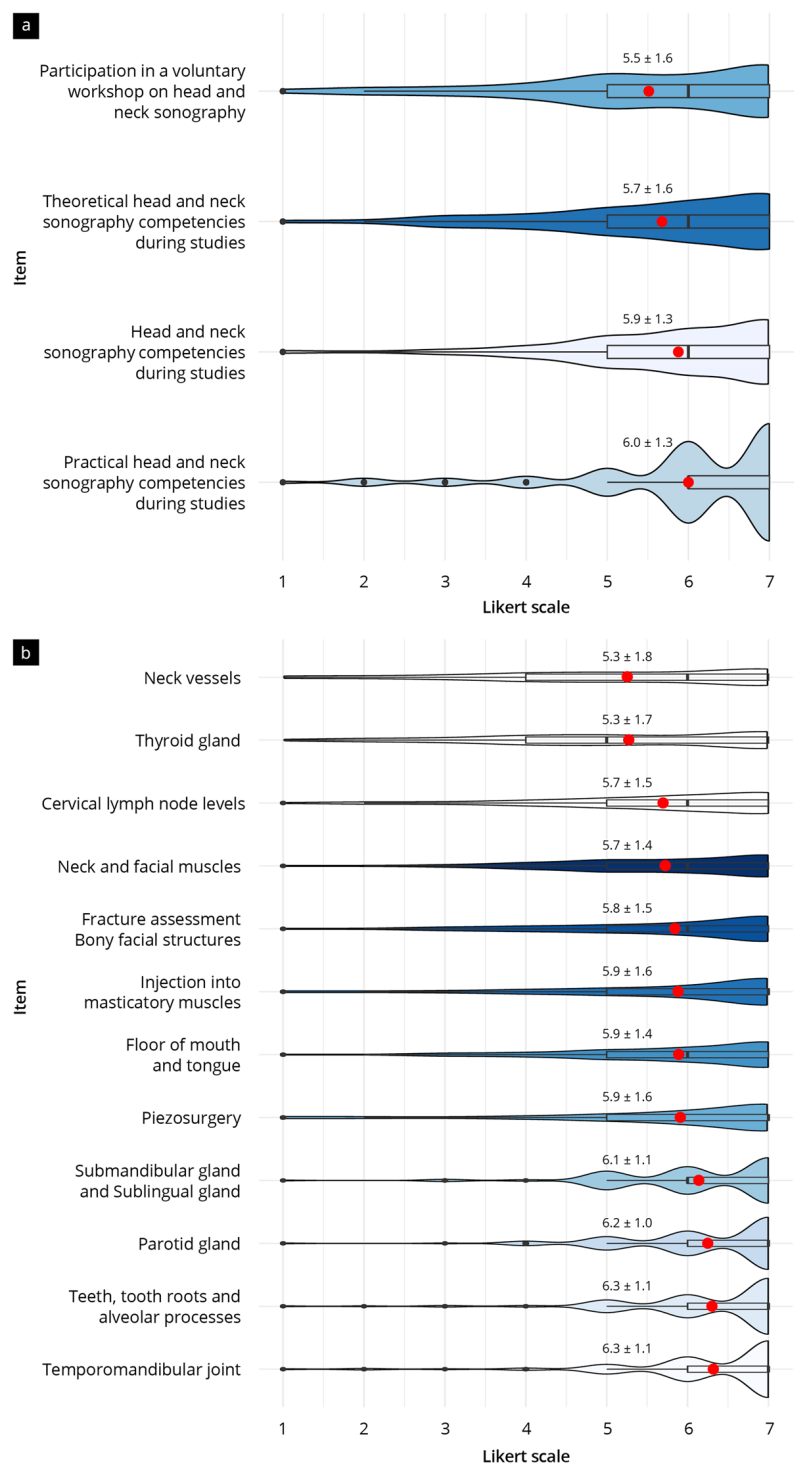
Results of questions regarding (**a**) the demand for and (**b**) the contents of undergraduate head and neck sonography training. Items are ranked from low to high demand

The responses to the questions regarding the demand for undergraduate ultrasound tuition are shown in Fig. 1a. Demand for head and neck sonography training was high in the cross-semester overall average (5.8 ± 1.3 scale points [SP]). The ratings of all subitems were, on average, in the range from 5.5 to 6.0 SP, without significant differences between the semesters (*p* = 0.06). Training in practical head and neck sonography skills was particularly important to the participants (6.0 ± 1.3 SP). Participants were not interested in working as peer tutors (3.5 ± 2.2 SP).

### Results of the topic inquiry and topic requests

Figure 1b and Supplements 3 and 4 show the desired topics of undergraduate head and neck sonography training. The most desired topics were “mandibular joint” (6.3 ± 1.1 SP), “teeth/roots/alveolar processes” (6.3 ± 1.1 SP), “parotid gland” (6.2 ± 1.0 SP), and “submandibular/sublingual glands” (6.1 ± 1.1 SP). Still highly desired, but to a lesser extent, were the topics “floor of the mouth/tongue” (5.9 ± 1.4 SP), “injection/infiltration masticatory muscles” (5.9 ± 1.6 SP), and “assessment of fractures of bony facial structures” (5.8 ± 1.5 SP). The “thyroid gland” (5.3 ± 1.7 SP) and “cervical blood vessels” (5.3 ± 1.8 SP) were significantly less desired than the other topics (*p* < 0.001).

### Survey reliability and validity

The reliability analysis demonstrated excellent internal consistency, with Cronbach’s Alpha values ranging from 0.93 to 0.95, confirming strong coherence among items. The KR-20 values ranged from 0.57 to 0.68, indicating acceptable reliability for dichotomous items, though slightly lower for Block 5.2 and 5.3. Construct validity was supported by high KMO values (Block 3: 0.83, Block 4: 0.90) and significant Bartlett’s tests (*p* < 0.001), confirming suitability for factor analysis. EFA identified a two-factor structure explaining 84% (Block 3) and 64% (Block 4) of the variance. Model fit indicators showed a TLI of 1.01 (Block 3), indicating an excellent fit, and 0.81 (Block 4), suggesting an acceptable but slightly suboptimal fit. The RMSEA for Block 4 was 0.16, indicating a moderate model fit. Item analysis confirmed high discrimination power, with all point-biserial correlations exceeding 0.65, ensuring that all items effectively differentiated between participants.

### Results of the survey on teaching methods and teaching materials

**Fig. 2:**
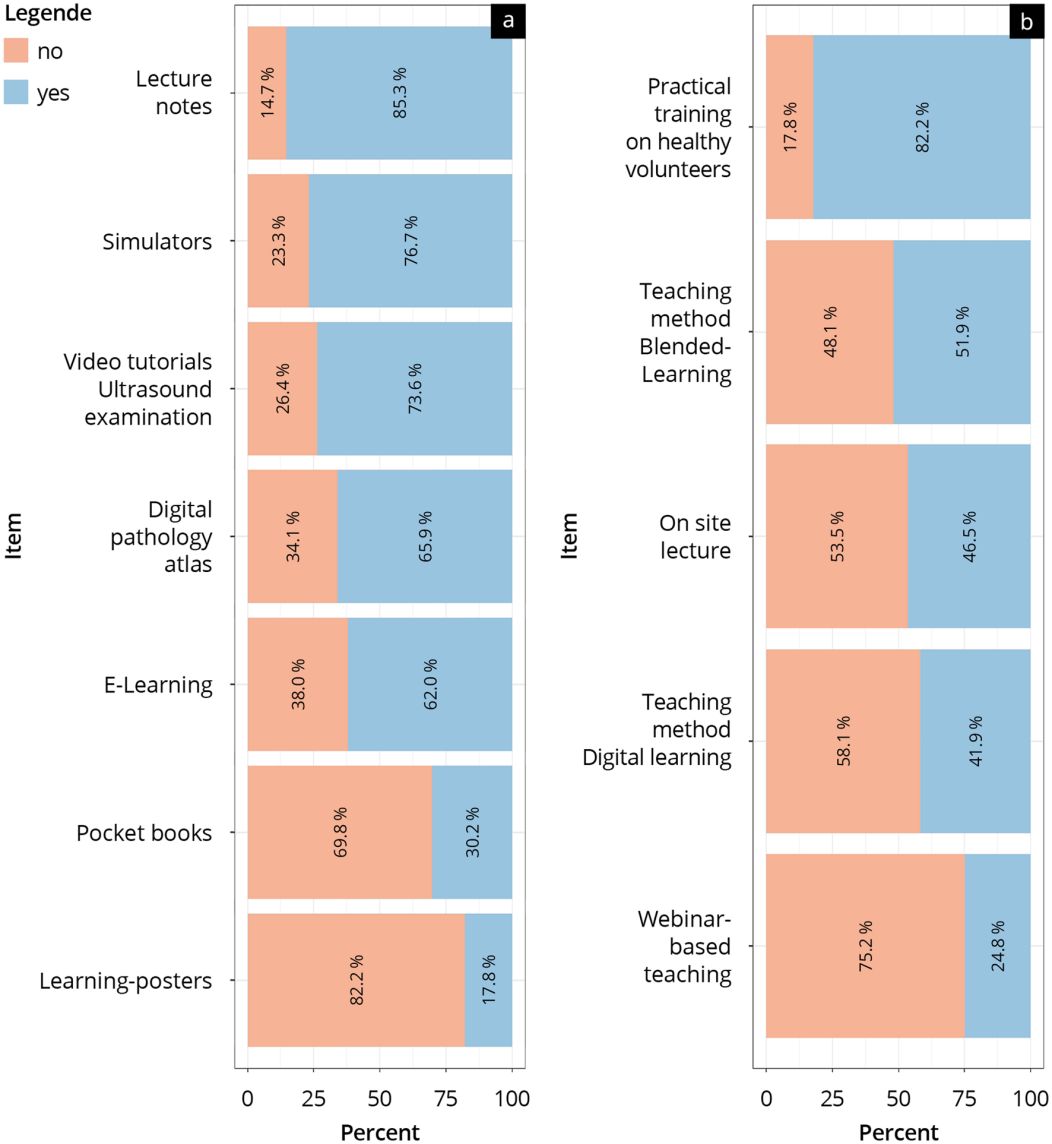
Preference of (**a**) study materials and (**b**) teaching methods of head and neck sonography training. Items are presented from high to low preference

Responses regarding preferred teaching materials and head and neck sonography training methods are shown in Fig. 2 and Supplement 5. The teaching methods “practical training on the subject” (82%) and “blended learning” (52%) were preferred most, while the use of “webinar apprentices” was significantly less desired (25%; *p* < 0.01). Of the study materials, “scripts” (85%), “simulators” (77%), “video instructions on ultrasound diagnostics” (74%), and “e-learning” (62%) were most preferred, while “learning posters” (18%) and “pocketbooks” (30%) were significantly less desired (*p* < 0.001).

### Influencing factors

Participants who had already observed or performed a head and neck sonography expressed a higher demand for training in head and neck sonography than those who had not observed or performed a head and neck sonography (*p* < 0.05).

## Discussion

### Relevance of the study and most important findings

Ultrasound is increasingly important in dentistry and maxillofacial surgery diagnostics [6–13, 16, 24, 42, 43]. It is essential for abscess differential diagnosis, helping general dentists distinguish abscesses from cysts and inflammatory swellings by assessing echotexture, vascularization, and fluid mobility. It enables the early detection of soft tissue infections around jawbones, aiding in identifying odontogenic abscesses and inflammatory changes. As a radiation-free, non-invasive imaging tool, sonography is a valuable alternative to conventional radiographic methods, particularly when evaluating submucosal infections and deep soft tissue involvement. By providing real-time imaging, sonography can support early diagnosis and treatment planning, including decisions on incision, drainage, or antibiotic therapy, improving clinical outcomes in general dental practice. In addition to classic transcervical sonography, intraoral techniques can be particularly useful in dentistry.

Currently, there are only a few corresponding training opportunities for dentistry students [20]. This represents a problem (“Problem Identification”) according to the Kern-SIX-Step-Approach [46]. Sonography training would be useful for several reasons: In the current version of the German National Competence-Based Catalogue of Learning Objectives for Dentistry [44, 45], sonography is explicitly mentioned as part of diagnostic imaging competencies. Dental students are expected to gain a basic understanding of various imaging modalities, including sonography, particularly in the context of head and neck diagnostics. The catalog emphasizes that future dentists should be able to recognize the indications, limitations, and potential applications of sonographic techniques, thereby underlining the growing relevance of ultrasound in modern dental education and practice. This curricular inclusion supports the need for structured introductory training in sonography during undergraduate studies.

Dentists are gatekeepers for oral cavity pathologies and guides to specialist care. They regularly examine the oral cavity, including the tongue and minor salivary glands, in symptomatic and asymptomatic patients. Sonography training could improve the diagnosis of conspicuous findings and address an unmet medical need.

This study is the first systematic examination exploring the demands and preferences of dentistry students for head and neck sonography training at a German university. The results underline the relevance of a specific curriculum in this area, addressing dentists’ specific requirements and interests. The curriculum should encompass key topics such as assessing the periodontal apparatus, temporomandibular joint, salivary glands, floor of the mouth, and tongue. Additionally, students preferred practical, hands-on training supported by complementary educational resources, including analog materials such as handouts or lecture notes and digital tools like video tutorials, e-learning modules, and blended learning. Such training should integrate simulations to enhance the learning experience as a bridge from theoretical knowledge to clinical application.

### Discussion of needs and topic priorities

Surveys of medical students have previously indicated a demand for integrating ultrasound teaching into the medical degree program [49, 50]. The results of the present study showed a high demand for practice-oriented ultrasound teaching among dentistry students during undergraduate studies, which similarly indicates a “positive” needs assessment [46]. This finding highlights the lack of structured ultrasound training in dental programs [20, 51]. Specific training addressing this gap in dentistry curricula would prepare dental students better for modern diagnostic challenges and purvey the capabilities and applications of sonography. Our survey showed that students who had already had contact with head and neck sonography had a significantly higher demand for more training in this field, illustrating the importance of early integration of appropriate content in undergraduate studies.

Based on this study’s survey of dentistry students, we can define a clear focus (“Goals and Objectives”) for a possible head-and-neck ultrasound training program [46]. Goals and objectives should focus on students acquiring sonographic representation of the teeth, the temporomandibular joint, the salivary glands, the neck levels, the floor of the mouth, and the tongue. These topics are relevant to interdisciplinary workflows [6–13, 16, 24, 42, 43]. Previously published head-and-neck ultrasound and training concepts for postgraduates [5, 29–35] and undergraduates in medicine [36–41] are similar to the desired topics.

Previous “Educational Strategies” in ultrasound training relied on analogous and digital study materials and teaching methods [19], whereby e-learning and blended learning are being integrated more recently [36, 37, 52–56]. Dentistry students preferred digital media, confirming the results of studies with medical students who also preferred blended learning and e-learning in head and neck ultrasound training [36, 37, 52, 57]. When implementing digital (e-learning) and analog (lecture note) study materials, an appealing and harmonized visual design increases the motivation for studying [58].

Dentistry students expressed a desire for practice-oriented ultrasound training, which coincides with survey results from other groups [49, 50, 59]. Head-and-neck ultrasound education should focus on training on probands, supplemented by simulator training [60–63] such as pig mandibles or virtual models [2, 64].

In addition to simulator-based teaching, video tutorials have emerged as a powerful educational tool [53] also in other medical fields, including sports and rehabilitation medicine. Expert-developed videos showcasing sonographic anatomy, scanning techniques, and pathological findings can significantly accelerate the learning curve for beginners by combining visual clarity with structured explanations. Especially mnemonic and metaphorical formats have shown promise in improving retention and comprehension of anatomical relationships and scanning protocols. In the future, scientific societies in dentistry should be encouraged to develop and disseminate high-quality, standardized ultrasound video tutorials to support structured training and knowledge transfer. This strategy could serve as a cost-effective, accessible complement to traditional teaching formats in dental ultrasound education [53, 65, 66].

“Peer tutors” are often used as teachers in student ultrasound training, which is evaluated very positively [19, 67]. In our survey, the participants’ interest in taking on a peer tutor role was low. This contrasts with surveys of medical students, who often report a higher willingness to engage as peer tutors in similar training settings. Dentistry students’ low interest in actively participating as peer tutors in ultrasound training should be evaluated before implementing a peer-based training curriculum [67].

The results of this study underscore a clear interest and perceived need among dental students for structured ultrasound training, suggesting a shift in awareness of sonography’s clinical relevance in dentistry. From a clinical perspective, sonography represents a valuable, radiation-free diagnostic tool that enables the evaluation of soft tissue structures, differentiation between abscesses, cysts, and inflammatory swellings, and assessment of peri-implant conditions. Integrating such training early in dental education may enhance students’ diagnostic skills, strengthen interdisciplinary collaboration, and contribute to more precise and patient-centered treatment planning. The demonstrated student demand underlines the potential of ultrasound to become a meaningful component of future dental practice and education.

### Assessment of questionnaire reliability and structural validity

The questionnaire exhibited excellent internal consistency, as reflected in Cronbach’s Alpha values above 0.90 across key subscales. This indicates that the items reliably measure coherent constructs related to ultrasound training in dental education. While the Kuder–Richardson Formula 20 (KR-20) values for certain dichotomous items were slightly lower, they remained within an acceptable range, likely reflecting natural variability in yes/no responses among students with differing levels of exposure and experience. The exploratory factor analysis supported the structural validity of the instrument, with a high proportion of explained variance and strong factor loadings, indicating that the items group together meaningfully. This confirms that the questionnaire captures distinct but theoretically consistent dimensions of students’ perceptions and expectations regarding ultrasound in dental education.

Furthermore, model fit indices such as the TLI and the RMSEA indicated an acceptable model fit. While the slightly elevated RMSEA suggests potential for refinement, particularly in item formulation or scale balance, it does not undermine the general validity of the instrument—especially considering the exploratory nature and relatively limited sample size of the study. The item discrimination analysis revealed that all items could differentiate between respondents with varying attitudes, such as interest in ultrasound and perceived relevance of sonography in clinical dental settings. This suggests that the questionnaire is sensitive to differing levels of engagement and experience among students and is thus suitable for broader applications in curriculum development.

These results confirm that the instrument is psychometrically sound and well-suited to assess dental students’ perspectives on ultrasound education. This provides a reliable empirical basis for evaluating training needs and informing educational policy. Moreover, the instrument’s strong performance supports its potential use in future multicenter or international studies, which could further enhance the generalizability of the findings and contribute to a standardized approach to integrating ultrasound into dental curricula.

### Future implementation and evaluation

This study does not address the implementation, evaluation, or subsequent revision and improvement steps of the Kern-cycle. Future evaluations should include investigating the subjective and objective theoretical and practical acquisition of competencies [19]. Theoretical examinations could consist of short-answer questions [68, 69], and practical exam formats could be performed as “direct observation of procedural skills” (DOPS) tests [34]. The interdisciplinary implementation of training with medical students should be considered as it offers several benefits. While interests may differ—medical students often show less interest in dental topics, and dentistry students may lack enthusiasm for areas like the thyroid or major neck vessels—joint training fosters interdisciplinary learning. Additionally, it provides an opportunity for exchange between disciplines, which is particularly valuable given the rarity of such interactions during the clinical study phase.

In the future, a curriculum could be implemented using a compact course format or a course format of several weeks [19, 38]. A curriculum could also include training in artificial intelligence and telemedicine [70, 71]. A stronger focus should be placed on integrating international approaches to further enhance and standardize ultrasound education in dental curricula.

While our study highlights a strong demand for ultrasound training among dental students, the implementation’s logistical and financial feasibility remains a critical consideration [72]. Successful integration of ultrasound into dental curricula requires addressing key factors such as equipment costs, which could be mitigated by sharing resources with other departments, and the availability of trained faculty, potentially facilitated by collaborations with radiology or medical ultrasound programs [73]. In addition, training time constraints must be balanced within existing curricula, and institutional barriers, including funding challenges and faculty acceptance, need to be considered [74]. Future research should further explore these aspects to ensure the practical feasibility of ultrasound education in dentistry.

Beyond theoretical instruction, practical simulation-based training, as already mentioned, is an essential component of ultrasound education, particularly for developing both diagnostic and interventional competencies. While hands-on cadaver training is often considered the gold standard, it poses several challenges, including high costs, limited availability, and technical demands related to specimen preservation. As a feasible and effective alternative, low-cost and bioanatomical phantoms—such as chicken breast, cheese, or gelatin-based models—have been successfully used in ultrasound workshops to teach needle guidance, aspiration techniques, fenestration, hydrodissection, and neural blocks. Incorporating such models into dental ultrasound training would allow students to acquire key skills safely, reproducibly, and cost-effectively. These practical approaches reflect the evolving role of ultrasound not only as a diagnostic tool but also as a means of guiding minimally invasive procedures in dental and maxillofacial practice [75].

### Limitations

This study has several limitations that should be acknowledged. The voluntary participation may have introduced self-selection bias, potentially overrepresenting students already interested in ultrasound. As most participants were in advanced semesters, their perceptions may not fully reflect those of students with less clinical exposure. Conducting the study at a single institution limits its external validity. While it identifies a training need, it does not comprehensively address logistical and financial barriers, such as equipment costs, faculty availability, and required training hours. The cross-sectional design prevents an analysis of long-term changes in student attitudes, and the small sub-sample sizes did not allow for subgroup analyses by semester. Although the questionnaire underwent reliability and validity testing, further refinements could strengthen its methodological robustness. For instance, the assessment of criterion validity was impossible at this juncture due to the absence of an external benchmark.

Nevertheless, subsequent research endeavors could encompass such a metric to facilitate validation. The study primarily reflects the German educational context, and integrating international perspectives could enhance its broader relevance. Future research should expand to multicenter and global studies with a more balanced semester representation to improve the generalizability and feasibility of implementing ultrasound training in dental curricula. The approach outlined in this study provides a foundation for these future investigations.

## Conclusion

In summary, this study emphasizes the demand among German undergraduate dentistry students for head and neck sonography training and provides a basis for developing and implementing a specific training curriculum. Blended learning, combining practical training with digital study materials and simulators, is highly desired. Future studies should concentrate on piloting a curriculum and evaluating its effects on dentistry students’ subjective and objective skills. In addition, multicentre studies and international cooperation are essential to developing standardized training content to meet dentistry’s diverse needs and current technical possibilities.

## Electronic supplementary material

Below is the link to the electronic supplementary material.

Supplementary Material 1

Supplementary Material 2

Supplementary Material 3

Supplementary Material 4

Supplementary Material 5

## Data Availability

Data cannot be shared publicly because of institutional and national data policy restrictions imposed by the Ethics committee since the data contain potentially identifying study participants’ information. Data are available upon request from the Johannes Gutenberg University Mainz Medical Center (contact via weimer@uni-mainz.de) for researchers who meet the criteria for access to confidential data (please provide the manuscript title with your inquiry).

## Abbreviations

Cherries: Checklist for Reporting Results of Internet E-Surveys
CT: Computed Tomography
DOPS: Direct Observation of Procedural Skills
DVT: Cone beam ct
EFA: Exploratory Factor Analysis
IQR: Interquartile Range
Kern-Cycle: Kern’s Six-Step Approach
KMO: Kaiser-Meyer-Olkin
KR-20: Kuder-Richardson Formula 20
MRI: Magnetic Resonance Imaging
QR: Code Quick Response Code
RMSEA: Root Mean Square Error of Approximation
SD: Standard Deviation
SP: Scale Points
TLI: Tucker-Lewis Index
