# Supplementary material for: Do dental students need sonography training? A prospective observational study
Source: BMC Med Educ. 2025 Apr 23;25:596. doi: 10.1186/s12909-025-07186-8 (PMC12020085; doi:10.1186/s12909-025-07186-8)
Supplement: Supplementary file 1 — Supplementary Material 1 [file 12909_2025_7186_MOESM1_ESM.pdf]

Supplement 1 Presentation of the needs analysis based on Checklist for Reporting Results of Internet E-Surveys (CHERRIES)

Item Category	Checklist Item	Explanation
**Design**	**Describe survey design**	Dentistry students of a German university of all semesters, all students were eligible to participate. It is not a convenience sample.
**Institutional Review Board (IBR) approval and informed consent process**	**IRB approval**	Application registered by the Ethics Commission of the Saarland Medical Association under the identification number 228/23.
	**Informed consent**	Consent to participate is requested at the beginning of the survey. By clicking a button, the participants may provide their responses to be used for scientific purposes.
	**Data protection**	Anonymous survey, no storage of personal data. Tracing responses to individuals is not possible.
**Development and pre-testing**	**Development and testing**	LimeSurvey Version 5.6.62 (LimeSurvey GmbH, Umfragedienste & Beratung, Papenreye 63, 22453 Hamburg, Deutschland)
		Development in interdisciplinary, interprofessional exchange (Otolaryngologists, didactics, statisticians, oral and maxillofacial surgeons, dentists, radiologists, dentistry students)
		Based on the current literature and learning objectives catalogues as well as recommendations from specialist societies
		Trial run with a small group (n = 10) of volunteers:<br>3 dentistry students, 2 dentists, 4 medical doctors, 2 medical students
		Optimization of the questionnaire/structure, spelling and functioning of test platform
**Recruitment process and description of the sample having access to the questionnaire**	**Open survey versus closed survey**	Open survey
	**Contact mode**	Via e-mail lists of the dean’s office, social media and during the university lectures in dentistry
	**Advertising the survey**	Advertising for participation in mails from the dean’s office, social media and in the university lectures in dentistry.
		Information text including advertising image as an attachment of an email or on social media as well as a presentation slide during lectures
		Access to survey via QR code or direct link
**Survey administration**	**Web/E-mail**	Data is automatically saved by survey programme, links to the query via e-mail, social media or as a QR code
	**Context**	Survey platform of the Center for Data Processing (ZDV) of the University of Mainz, publication/access only via Link/QR code
		No posting/publication on a website
	**Mandatory/voluntary**	voluntary

Item Category	Checklist Item	Explanation
	**Incentives**	The development of a specific teaching concept addressing the wishes of the students. No individual advantages/disadvantages by deciding for or against participation.
	**Time/Date**	From 12th December 2023 to 09th February 2024
	**Randomization of items or questionnaires**	No Randomisierung
	**Adaptive questioning**	No
	**Number of Items**	Page 1: Start
		Page 2: 8 questions
		Page 3: 2 questions
		Page 4: 6 questions
		Page 5: End
	**Number of screens (pages)**	4
	**Completeness check**	All questions mandatory to complete the survey
		Answer option “not applicable” for all questions
	**Review step**	„back” button and participants may print out their answers or save as a file
**Response rates**	**Unique site visitor**	IP was not recorded
	**View rate (Ratio of unique survey visitors/unique site visitors)**	Not relevant as site was only used for survey
	**Participation rate (Ratio of unique visitors who agreed to participate/unique first survey page visitors)**	Total: 131
	**Completion rate (Ratio of users who finished the survey/users who agreed to participate)**	2 incomplete surveys:
		2 incomplete, but did send
		129 complete
		Ratio: 165:190=98%
**Preventing multiple entries from the same individual**	**Cookies used**	Cookies were set, to avoid multiple completions by one user
	**IP check**	https://www.bfdi.bund.de/SharedDocs/Pressemitteilungen/DE/2017/11_BGH_Breyer.html
		Were not recorder because of the General Data Protection Regulation (GDPR)

Item Category	Checklist Item	Explanation
	**Log file analysis**	only Cookies
	**Registration**	Open survey without saving for participants
	**Handling of incomplete questionnaires**	Yes, only fully completed questionnaires were analysed
	**Questionnaires submitted with an atypical timestamp**	All questionnaires were included regardless of the processing time
	**Statistical correction**	No
