# Supplementary material for: Do dental students need sonography training? A prospective observational study
Source: BMC Med Educ. 2025 Apr 23;25:596. doi: 10.1186/s12909-025-07186-8 (PMC12020085; doi:10.1186/s12909-025-07186-8)
Supplement: Supplementary file 2 — Supplementary Material 2 [file 12909_2025_7186_MOESM2_ESM.pdf]

Supplement 2 Requirements and needs analysis questionnaire

1. General information/ personal data

1.1. At which **university** are you studying?
____

1.2. How **old** are you? ________________ years

1.3. What **gender** are you?

male ☐ female ☐ divers☐

1.4. In which **semester** are you currently? __________________semester

2. Previous experiences in imaging procedures

2.1. Which of the following imaging procedures have you already been in contact with during your studies? (multiple choice possible)

☐	2.1.1	X-ray
☐	2.1.2	Computer tomography (CT)
☐	2.1.3	Magnetic resonance imaging (MRI)
☐	2.1.4	Ultrasound
☐	2.1.5	Cone beam CT (CBCT)
☐	2.1.6	other_________________

2.2. Have you already **viewed a head and neck ultrasound** examination? (Please select only one of the following answers)

yes☐ no ☐

2.3. Have you already **performed a head and neck ultrasound** examination? (Please select only one of the following answers)

yes☐ no ☐

3. Interest of integration of ultrasound diagnostics in the degree program

		not at all very high<br>1 7
**3.1**	Are you interested in learning **theoretical diagnostic** head and neck ultrasound skills during your studies?	☐ ☐ ☐ ☐ ☐ ☐ ☐
**3.2**	Are you interested in learning **practical diagnostic** head and neck ultrasound skills during your studies?	☐ ☐ ☐ ☐ ☐ ☐ ☐
**3.3**	Are you interested in learning **diagnostic** head and neck ultrasound skills during your studies?	☐ ☐ ☐ ☐ ☐ ☐ ☐
**3.4**	Would you take a **voluntary elective/workshop** to acquire these skills?	☐ ☐ ☐ ☐ ☐ ☐ ☐
**3.5**	Would you be interested in working as a **tutor** in a student ultrasound course?	☐ ☐ ☐ ☐ ☐ ☐ ☐

4. Topics of an ultrasound training

Which of the following **topics** should be included in a possible **elective/workshop** on **head and neck sonography** for **dental students**?

		No interest very high all interest at 1 7					
**4.1**	Sonoanatomy and pathology of the **teeth, teeth roots and alveolar processes**?	☐	☐	☐	☐	☐	☐
**4.2**	Sonoanatomy and **fracture evaluation** of facial bones (e.g. orbital floor or zygomatic arch)?	☐	☐	☐	☐	☐	☐
**4.3**	**Therapeutic ultrasound**: Piezosurgery, maybe practice on pig jaws?	☐	☐	☐	☐	☐	☐
**4.4**	Sonoanatomy and pathology of the **muscles** of the neck and the face?	☐	☐	☐	☐	☐	☐
**4.5**	**Ultrasound-guided injection in the face** or the facial/chewing muscles, e.g. botolinum toxin (Botox)?	☐	☐	☐	☐	☐	☐
**4.6**	Sonoanatomy and pathology **of the cervical neck vessels**?	☐	☐	☐	☐	☐	☐
**4.7**	Sonoanatomy and pathology of the **thyroid gland**?	☐	☐	☐	☐	☐	☐
**4.8**	Sonoanatomy and pathologies of the cervical **neck levels** (lymph node stations -> frequent metastatic routes of tumors in the maxillofacial region)?	☐	☐	☐	☐	☐	☐
**4.9**	Sonoanatomy and pathology of the **floor of the mouth** and the **tongue**?	☐	☐	☐	☐	☐	☐
**4.10**	Sonoanatomy and pathology of the **temporomandibular joint**?	☐	☐	☐	☐	☐	☐
**4.11**	Sonoanatomy and pathology of the **parotid gland**?	☐	☐	☐	☐	☐	☐
**4.12**	Sonoanatomy and pathology of the **submandibular and sublingual gland**?	☐	☐	☐	☐	☐	☐

You can enter additional topic suggestions in the following field:

5. teaching methods and study materials/media

		not at all completely<br>1 7
**5.1.**	To which extent do you agree with the statement that **blended learning concepts** (combination of digital teaching methods, e.g. e-learning with classroom teaching methods/on-site teaching) should be used in ultrasound training **to teach new skills**?	☐ ☐ ☐ ☐ ☐ ☐ ☐

5.2. Which of the following **teaching materials** should be used as part of an ultrasound course? (multiple choice possible)

☐	5.2.1	lecture notes
☐	5.2.2	e-learning
☐	5.2.3	digital pathology atlas
☐	5.2.4	Video instructions for an ultrasound examination (transducer guidance etc.)
☐	5.2.5	educational poster
☐	5.2.6	pocket books or cards
☐	5.2.7	simulators

5.3 Which of the following **teaching methods** should be used as part of an ultrasound course? (multiple choice possible)

☐	5.3.1	blended learning (combination of digital teaching methods and traditional classroom teaching)
☐	5.3.2	digital learning
☐	5.3.3	practical training on healthy volunteers
☐	5.3.4	webinar based teaching
☐	5.3.5	on site lecture

6. general feedback

If you have any suggestions on the issue of diagnostic ultrasound/sonography in dental studies or general feedback on the subject, please feel free to let us know.
