# Supplementary material for: Do dental students need sonography training? A prospective observational study
Source: BMC Med Educ. 2025 Apr 23;25:596. doi: 10.1186/s12909-025-07186-8 (PMC12020085; doi:10.1186/s12909-025-07186-8)
Supplement: Supplementary file 3 — Supplementary Material 3 [file 12909_2025_7186_MOESM3_ESM.pdf]

Supplement 3: Comparison of the ratings with regard to the subject wishes of a head and neck sonography; the table shows the p-values of the difference test

	Teeth, tooth roots, alveolar processes	Temporomandibular joint	Parotid gland	Submandibular gland and sublingual gland	Floor of mouth and tongue	Injection into masticatory muscles	Piezo-surgery	Bony facial structures	Neck and facial muscles	Cervical lymph node levels	Thyroid gland	Neck vessels
**Teeth, tooth roots, alveolar processes**	/	0.91	0.68	0.22	0.01	0.01	0.02	<0.001	<0.001	<0.001	<0.001	<0.001
**Temporomandibular joint**	0.91	/	0.60	0.19	0.01	0.01	0.02	<0.001	<0.001	<0.001	<0.001	<0.001
**Parotid gland**	0.68	0.60	/	0.41	0.02	0.03	0.04	0.01	<0.001	<0.001	<0.001	<0.001
**Submandibular gland and sublingual gland**	0.22	0.19	0.41	/	0.11	0.13	0.17	0.07	0.01	0.01	<0.001	<0.001
**Floor of mouth and tongue**	0.01	0.01	0.02	0.11	/	0.97	0.93	0.79	0.33	0.28	<0.001	<0.001
**Injection into masticatory muscles**	0.01	0.01	0.03	0.13	0.97	/	0.91	0.84	0.38	0.33	<0.001	<0.001
**Piezosurgery**	0.02	0.02	0.04	0.17	0.93	0.91	/	<0.001	0.32	0.28	<0.001	<0.001
**Bony facial structures**	<0.001	<0.001	0.01	0.07	0.79	0.84	0.00	/	0.49	0.43	<0.001	<0.0010
**Neck and facial muscles**	<0.001	<0.001	<0.001	0.01	0.33	0.38	0.32	0.49	/	0.90	0.02	0.02
**Cervical lymph node levels**	<0.001	<0.001	<0.001	0.01	0.28	0.33	0.28	0.43	0.90	/	0.03	0.03
**Thyroid gland**	<0.001	<0.001	<0.001	<0.001	<0.001	<0.001	<0.001	<0.001	0.02	0.03	/	0.94
**Neck vessels**	<0.001	<0.001	<0.001	<0.001	<0.001	<0.001	<0.001	<0.001	0.02	0.03	0.94	/
