# Supplementary material for: Do dental students need sonography training? A prospective observational study
Source: BMC Med Educ. 2025 Apr 23;25:596. doi: 10.1186/s12909-025-07186-8 (PMC12020085; doi:10.1186/s12909-025-07186-8)
Supplement: Supplementary file 4 — Supplementary Material 4 [file 12909_2025_7186_MOESM4_ESM.pdf]

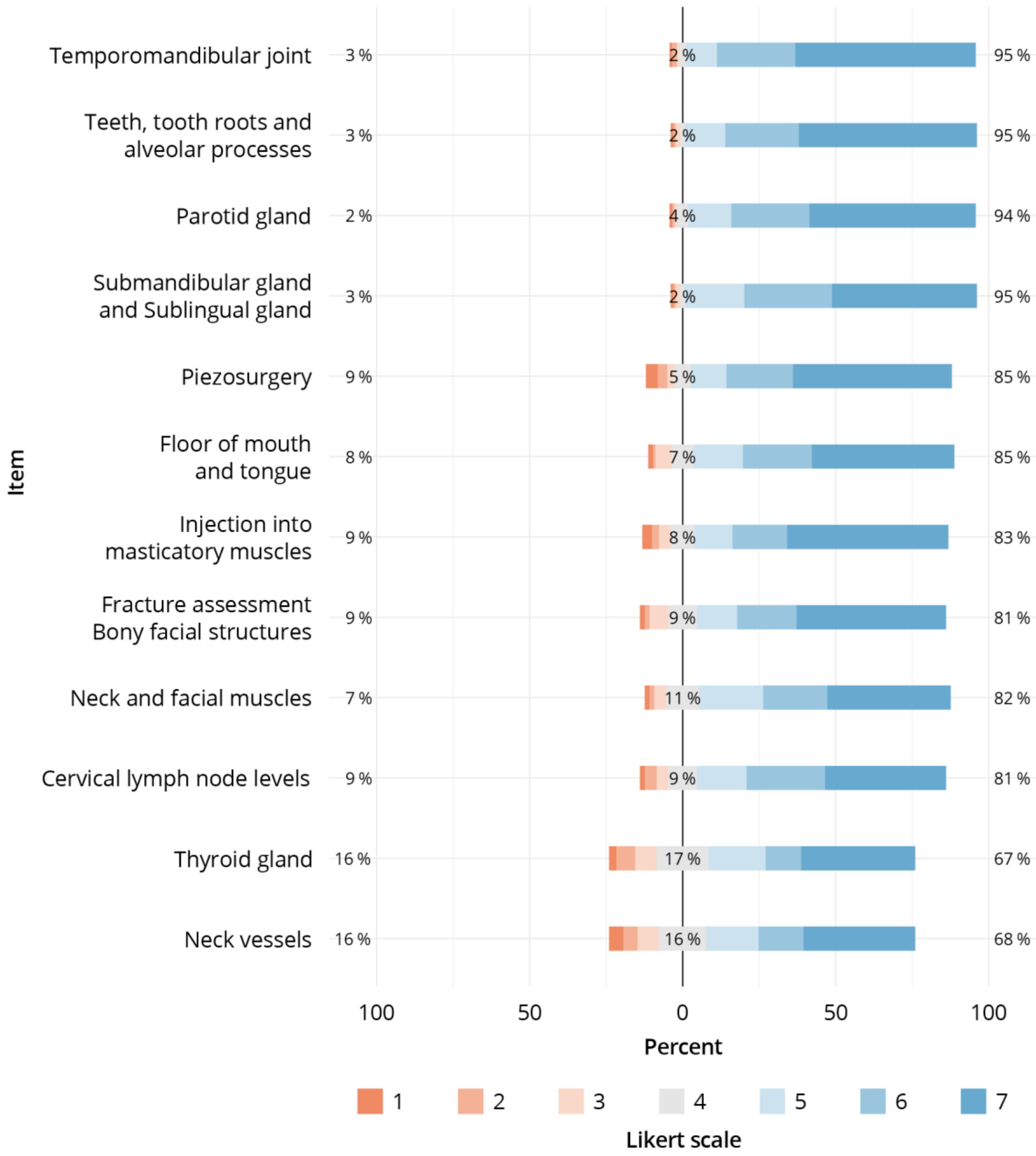

Supplement 4 Responses regarding the desired topics of a head-neck sonography training. The percentage of the proportion of responses per level oof the 7-level Likert answering format ranging from 1 = “not at all” and 7 = “full and entirely”. Items are ordered from high to low preference.
