# Supplementary material for: Do dental students need sonography training? A prospective observational study
Source: BMC Med Educ. 2025 Apr 23;25:596. doi: 10.1186/s12909-025-07186-8 (PMC12020085; doi:10.1186/s12909-025-07186-8)
Supplement: Supplementary file 5 — Supplementary Material 5 [file 12909_2025_7186_MOESM5_ESM.pdf]

Supplement 5: Comparison of the evaluations regarding the teaching materials and teaching methods of a head and neck sonography; the table shows the p-values of the difference test

Teaching materials							
	Lecture notes	Simulator	Video tutorials, Ultrasound examination	Digital pathology atlas	E-Learning	Pocket books	Learning-posters
**Lecture notes**	/	0.081	0.02	<0.001	<0.0001	<0.001	<0.001
**Simulators**	0.08	/	0.56	0.05	0.01	<0.001	<0.001
**Video tutorials, Ultrasound examination**	0.02	0.56	/	0.18	0.05	<0.001	<0.001
**Digital pathology atlas**	<0.0001	0.05	0.18	/	0.52	<0.001	<0.001
**E-Learning**	<0.0001	0.01	0.05	0.52	/	<0.001	<0.001
**Pocket books**	<0.001	<0.001	<0.001	<0.001	<0.001	/	0.02
**Learning-posters**	<0.001	<0.001	<0.001	<0.001	<0.001	0.02	/

Teaching methods					
	Practical training on healthy volunteers	Blended learning	On site lecture	Digital learning	Webinar-based teaching
**Practical training on healthy volunteers**	/	<0.001	<0.001	<0.001	<0.001
**Blended learning**	<0.001	/	0.38	0.10	<0.0001
**On site lecture**	<0.001	0.38	/	0.45	<0.0001
**Digital learning**	<0.001	0.10	0.45	/	<0.0001
**Webinar-based teaching**	<0.001	<0.00001	<0.001	<0.01	/
